# Supplementary material for: Implementation of maternal and perinatal death reviews: a scoping review protocol
Source: BMJ Open. 2019 Nov 27;9(11):e031328. doi: 10.1136/bmjopen-2019-031328 (PMC6886965; doi:10.1136/bmjopen-2019-031328)
Supplement: Supplementary data [file bmjopen-2019-031328supp001.pdf]

Supplementary Files for *Implementation of maternal and perinatal death reviews: a scoping review*

SUPPLEMENTARY FILE 1

Table S1.1: Frameworks considered when developing the conceptual frameworks for the scoping review

Framework	Level	Characteristic	Pros - why we considered it	Cons – why we decided not to use it
Context and Implementation of Complex Intervention - CICI Framework (Pfadenhauer) [1]	Macro, Meso, Micro	Comprises three dimensions which interact: Context (i.e., geographical, epidemiological, socio-cultural, socio-economic, ethical, legal, political); implementation (i.e., implementation theory, process, strategies, agents and outcomes); setting (specific physical location).	A determinant framework that seeks to conceptualise, describe and understand the multiple influences on implementation outcomes; Enables understanding of implementation process at multiple levels across different factors; Provides data extraction tools for qualitative and quantitative reviews	It focuses on the operationalization of context, setting and implementation *not on the intervention itself.* Yet we wanted to understand more about the how the intervention is working and being implemented.
Dynamic sustainability framework (Chambers) [2]	Macro, Meso,	Considers the intervention, the context and the broader ecological system over time	A new paradigm to consider the long-term use and ongoing improvement of interventions; posits that ongoing QI is the aim	Does not provide enough focus on the implementation process now – rather would allow for exploring implementation of audit over time to see if process leads to change (which is not purpose of scoping review)
Promoting Action on Research Implementation in Health Services - PARiHS (Kitson) [3]	Meso	Considers the interactions between three key elements: Evidence, Context, Facilitation	It argues that successful implementation (SI) of evidence into practice had as much to do with the context or setting where the new evidence was being	Focuses on knowledge translation component of implementation. Many of the constructs do not relate to what we think would be needed to

			introduced and how it was introduced	understand the MPDSR process
Consolidated Framework for Implementation Research - CFIR (Damschroder) [4]	Macro, Meso, Macro	Comprises five major domains and considers their interactions: the intervention, inner and outer setting, the individuals involved, and the process by which implementation is accomplished	Enables understanding of different levels and different factors that influence implementation; it includes the intervention outcome but also allows for understanding process as well as influencing factors at different levels	Not all constructs are applicable to MPDSR implementation and some elements of what we seek to understand might be missing
Normalization Process Theory (May) [5]	Meso, Micro	Comprises four constructs: Coherence, Cognitive Participation, Collective Action, and Reflexive Monitoring	it explores the fluid, dynamic and interactive processes between context, actors and objects<br>Investigates the process by which an intervention becomes embedded	Does not include enough factors that should be considered for understanding implementation factors (focuses only on meso, micro level)

SUPPLEMENTARY FILE 2: SEARCH STRATEGY

Search terms used for search engines from January 2004 to July 2018:

("maternal mortality" OR "perinatal death" OR "maternal death" OR "perinatal mortality" OR "fetal mortality" OR "stillbirth") **AND** (“audit” OR "surveillance and response")

Table 2.1 Concept and search terms included

Concept	Search terms
Maternal & Perinatal death	maternal mortality, maternal death, stillbirth, fetal mortality, perinatal death, perinatal mortality
Audit	Audit, surveillance and response

Kinney MV, *et al. BMJ Open* 2019; 9:e031328. doi: 10.1136/bmjopen-2019-031328

SUPPLEMENTARY FILE 3: DRAFT DATA EXTRACTION FORM

	Reviewer assigned	
	Inclusion	Yes<br>No
**Reference details**	Ref ID	
	Author	
	Title	
	Year	
	Abstract	
	Journal	
	Volume	
	Issue	
	Pages	
	URL	
	Source	Database<br>Online search<br>Consultation
Background to reference	Country of first affiliation	
	Country of Study	Name country<br>Multiple<br>N/A
	Country Setting	Upper MI<br>Lower MI<br>LIC<br>LMIC<br>Not specified (add to notes)
	Region of World	Sub-Saharan Africa<br>East Asia & Pacific<br>Latin America & Caribbean<br>South Asia<br>Europe & Central Asia<br>Middle East & North Africa<br>International
	Organization Type of authors	Government<br>NGO<br>University/academic institution<br>Independent<br>Mixed including government<br>Mixed not including government<br>Other (add note)
	Funder type (sector)	Government<br>Multilateral (e.g. WHO, World Bank, UN)<br>Bilateral (e.g. USAID, DIFD, IDRC)<br>Foundation (e.g. BMGF, Rockefeller)<br>Mixed includes government

		Mixed does not include government<br>No funding received
	Notes	
Content of reference	Focus of the audit	Maternal & perinatal<br>Maternal<br>Perinatal
	Notes	
	History/timeline	Yes<br>No
	Notes	
	Scale	National<br>Subnational<br>Selected Facilities<br>Combination Of Levels<br>Global<br>Multicountry<br>Other
	Notes	
Reference type	Document type	Academic journal article<br>Academic journal commentary<br>Academic review<br>Grey lit online<br>Grey lit not online<br>other
	Methods section	Yes<br>No
	Research Design	Quantitative<br>Qualitative<br>Mixed<br>NA
	Level of Study	Macro<br>Meso<br>Micro<br>Combination
	Notes	
Domain 1: Intervention/MPDSR	Executing audit	Described<br>Not described
	Notes	
	Cost & funding	Described<br>Not described
	Notes	
	Intervention source	Described<br>Not described
	Notes	
	Evidence strength & quality	Described<br>Not described

	Notes	
	Relative advantage	Described<br>Not described
	Notes	
	Trialability	Described<br>Not described
	Notes	
	Reflectivity	Described<br>Not described
	Notes	
	Adaptability	Described<br>Not described
	Notes	
	Complexity	Described<br>Not described
	Notes	
Domain 2: Outer Setting	Policy and planning	Described<br>Not described
	Notes	
	Resource flows	Described<br>Not described
	Notes	
	Linkages to other actors	Described<br>Not described
	Notes	
	Pressure	Described<br>Not described
	Notes	
	Community links	Described<br>Not described
	Notes	
	Cosmopolitanism	Described<br>Not described
	Notes	
Domain 3: Inner Setting	Readiness to implement	Described<br>Not described
	Notes	
	Structural characteristics of social architecture	Described<br>Not described
	Notes	
	Incentive/rewards	Described<br>Not described
	Notes	
	Networks & communication	Described<br>Not described

Kinney MV, *et al. BMJ Open* 2019; 9:e031328. doi: 10.1136/bmjopen-2019-031328

	Notes	
	Culture	Described<br>Not described
	Notes	
	Implementation climate	Described<br>Not described
	Notes	
	Agents of change	Described<br>Not described
	Notes	
Domain 4: Individuals	Tech skills & knowledge	Described<br>Not described
	Notes	
	Individual motivation, Self-efficacy	Described<br>Not described
	Notes	
	Individual commitment	Described<br>Not described
	Notes	
	Individual orientation	Described<br>Not described
	Notes	
	Individual state of change	Described<br>Not described
	Notes	

REFERENCES

1. Pfadenhauer LM, Gerhardus A, Mozygemba K, Lysdahl KB, Booth A, Hofmann B et al. Making sense of complexity in context and implementation: the Context and Implementation of Complex Interventions (CICI) framework. Implement Sci. 2017; 12(1):21.
2. Chambers DA, Glasgow RE,Stange KC. The dynamic sustainability framework: addressing the paradox of sustainment amid ongoing change. Implement Sci. 2013; 8:117.
3. Kitson AL, Rycroft-Malone J, Harvey G, McCormack B, Seers K,Titchen A. Evaluating the successful implementation of evidence into practice using the PARiHS framework: theoretical and practical challenges. Implement Sci. 2008; 3:1.
4. Damschroder LJ, Aron DC, Keith RE, Kirsh SR, Alexander JA,Lowery JC. Fostering implementation of health services research findings into practice: a consolidated framework for advancing implementation science. Implement Sci. 2009; 4:50.
5. Murray E, Treweek S, Pope C, MacFarlane A, Ballini L, Dowrick C et al. Normalisation process theory: a framework for developing, evaluating and implementing complex interventions. BMC medicine. 2010; 8:63.
