# Supplementary material for: Implementation of maternal and perinatal death reviews: a scoping review protocol
Source: BMJ Open. 2019 Nov 27;9(11):e031328. doi: 10.1136/bmjopen-2019-031328 (PMC6886965; doi:10.1136/bmjopen-2019-031328)
Supplement: Author's manuscript [file bmjopen-2019-031328.draft_revisions.pdf]

BMJ Open

BMJ Open is committed to open peer review. As part of this commitment we make the peer review history of every article we publish publicly available.

When an article is published we post the peer reviewers' comments and the authors' responses online. We also post the versions of the paper that were used during peer review. These are the versions that the peer review comments apply to.

The versions of the paper that follow are the versions that were submitted during the peer review process. They are not the versions of record or the final published versions. They should not be cited or distributed as the published version of this manuscript.

BMJ Open is an open access journal and the full, final, typeset and author-corrected version of record of the manuscript is available on our site with no access controls, subscription charges or pay-per-view fees (http://bmjopen.bmj.com).

If you have any questions on BMJ Open's open peer review process please email info.bmjopen@bmj.com

BMJ Open

# Implementation of maternal and perinatal death reviews: a scoping review


| | |
|---|---|
| Journal: | *BMJ Open* |
| Manuscript ID | bmjopen-2019-031328 |
| Article Type: | Protocol |
| Date Submitted by the Author: | 28-Apr-2019 |


| | |
|---|---|
| Complete List of Authors: | Kinney, Mary; University of the Western Cape Faculty of Community and Health Sciences, School of Public Health<br>Walugembe, David; University of Western Ontario, School of Health Studies and Faculty of Information and Media Studies<br>Waiswa, Peter; Makerere University College of Health Sciences, Schollo of Public health; Karolinska Institutet,<br>Wanduru, Phillip; Makerere University<br>George, Asha S. ; University of the Western Cape, |


| | |
|---|---|
| Keywords: | Maternal medicine < OBSTETRICS, Clinical audit < HEALTH SERVICES ADMINISTRATION & MANAGEMENT, Surveillance, implementation, Quality in health care < HEALTH SERVICES ADMINISTRATION & MANAGEMENT, perinatal |


SCHOLARONE™
Manuscripts

**Title**: Implementation of maternal and perinatal death reviews: a scoping review

**Authors**: Mary V. Kinney, David Roger Walugembe, Phillip Wanduru, Peter Waiswa, Asha George

Affiliations:

- Mary V. Kinney, School of Public Health, University of the Western Cape, Private Bag X17, Bellville 7535 South Africa; email: mkinney@uwc.ac.za; telephone: (+27) 834448211
- David Roger Walugembe, School of Health Studies and Faculty of Information and Media Studies, The University of Western Ontario, London, ON, Canada
- Phillip Wanduru, College of Health Sciences, Makerere University, Kampala, Uganda
- Peter Waiswa, College of Health Sciences, Makerere University School of Public Health, Kampala, Uganda
- Asha George, School of Public Health, University of the Western Cape, Bellville, South Africa

Word count paper: 3025/4000

For peer review only



For peer review only - http://bmjopen.bmj.com/site/about/guidelines.xhtml

**ABSTRACT**

## Introduction

Maternal and perinatal death surveillance and response (MPDSR), or any related form of audit, is a quality improvement mechanism used to prevent future maternal and perinatal deaths. While the existence of MPDSR policies is routinely measured, measurement and understanding of policy implementation has lagged behind. In this paper, we present a theory-based conceptual framework for understanding MPDSR implementation as well as a scoping review protocol to understand factors influencing MPDSR implementation in low-and middle-income countries (LMICs).

## Methods and analysis

The Consolidated Framework for Implementation Research will inform the development of a theory-based conceptual framework for MPDSR implementation. The methodology for the scoping review will be guided by an adapted Arksey and O'Malley approach. Documents will include published and grey literature sourced from electronic databases (PubMed, CINAHL, SCOPUS, Web of Science, JSTOR, LILACS), the WHO Library, Maternal Death Surveillance and Response Action Network, Google, the reference lists of key studies, and key experts. Two reviewers will independently screen titles, abstracts and full studies for inclusion. All discrepancies will be resolved by an independent third party. We will include studies published in English from 2004 to July 2018 that present results on factors influencing implementation of MPDSR, or any related form. Qualitative content and thematic analysis will be applied to extracted data according to the theory-based conceptual framework. Stakeholders will be consulted at various stages of the process.

## Dissemination and Ethics

The scoping review will synthesise implementation factors relating to MPDSR in LMIC as described in the literature. This review will contribute to the work of the Countdown to 2030 Drivers Group, which seeks to explore key contextual drivers for equitable and effective coverage of maternal and child health interventions. Ethics approval is not required. The results will be disseminated through various channels, including a peer-reviewed publication.

**STRENGTHS AND LIMITATIONS OF THE STUDY**

- This will be the first scoping review to identify and describe implementation factors relating to maternal and perinatal death surveillance and response (MPDSR) in low and middle-income countries.
- The search strategy includes six electronic databases with peer-reviewed literature as well as three online search engines to identify published and grey literature including academic research articles, commentaries, other related reviews, and reports.
- The results will be presented using an adapted theory-based conceptual framework for MPDSR implementation, and data analysis will involve qualitative thematic and content analysis linked to this framework.
- Stakeholders including the World Health Organization's MPDSR Technical Working Group as well as the Countdown to 2030 Drivers Technical Working Group will be consulted and engaged throughout the study review process.



For peer review only - http://bmjopen.bmj.com/site/about/guidelines.xhtml

For peer review only



For peer review only - http://bmjopen.bmj.com/site/about/guidelines.xhtml

## INTRODUCTION

Improving quality of care is a global health imperative for achieving the Sustainable Development Goal for health. There has been a specific focus on improving facility-based maternal and newborn care,[1] as evidenced by the release of specific World Health Organization standards and the launch of a new Quality of Care network to support implementation in nine countries in South Asia and Africa.[2 3] With rising attention on quality of care initiatives, [4-7] more needs to be understood about their implementation, including for audit and feedback.[8]

Quality improvement efforts are ongoing processes, which are often considered complex, fluid and context specific.[9-12] A variety of factors,[9 12-17] including context,[18] can influence implementation of these types of interventions. Implementation theory enables more complex interventions to be unpacked and examined.[9 12 14-17] This approach allows for exploration of issues, such as trust, credibility, relationships and hierarchies to understand factors that support or hinder implementation.[19]

One important quality improvement approach is maternal death surveillance and response (MDSR), perinatal death audit, or a joint maternal and perinatal death surveillance and response (MPDSR).[4 20 21] Maternal and/or perinatal death surveillance and response, referred to in this paper as M/PDSR, is an established mechanism to examine the circumstances surrounding each death to prevent future deaths.[22] It requires continuous application of monitoring-review-act cycles[10] to capture information on the number and causes of deaths, with systematic, critical analysis of the quality of care received for a sample of or for all cases, in a no-blame, interdisciplinary setting, with a view to improving the care provided to all mothers and babies.[23]

In the past fifteen years, there has been momentum to strengthen clinical audit practice for maternal and perinatal death as part of quality improvement activities,[24-27] including the development of global technical guidelines.[23 28] Many low- and middle-income countries (LMIC) have adopted national guidelines, however few have robust MPDSR systems.[29] A growing number of studies have investigated the implementation of M/PDSR in selected countries, and some reviews have explored implementation factors for maternal death reviews or perinatal death audits, separately. For example, a structured literature review of accountability mechanisms for maternal and newborn health in sub-Saharan Africa found MDSR the most common mechanism for performance accountability.[30] A systematic review of facility-based perinatal mortality audit in LMIC countries in 2009 identified ten low-quality evaluations with mortality outcome data.[31] A literature review conducted in 2015 on facility based perinatal audits explored enablers and barriers according to the health system building blocks.[29] While valuable contributions to the literature, these previous reviews did not considered implementation theory to assess implementation of M/PDSR nor of the full range of types of maternal and/or perinatal death reviews.[29 30 32]

### Study rationale

Global agencies, such as the World Health Organization, have created guidelines for M/PDSR and are encouraging LMIC to move forward with implementation.[23 26] Further understanding of the enablers and barriers of implementation in LMIC is needed to support roll out of this intervention across and within countries. A rigorous, scoping review has not yet been undertaken to map publications in LMIC on factors influencing implementation of M/PDSR in ways that are inclusive of either maternal and/or perinatal death audits.



For peer review only - http://bmjopen.bmj.com/site/about/guidelines.xhtml

## Study Objectives

To map and synthesize the available literature on the factors that support or hinder M/PDSR implementation using a theory-based conceptual implementation framework. We will also explore common, if any, implementation factors between MDSR, PDSR or MPDSR.

# METHODS AND ANALYSIS

## Conceptual model

This section of the protocol presents a proposed theory-based conceptual framework, which will be pilot tested and adapted for the data extraction and analysis. To develop the framework, we considered conceptualization of the M/PDSR as an intervention process and reviewed various theory-based implementation frameworks.

### Conceptualizing M/PDSR

M/PDSR is a continuous action cycle for quality improvement that links maternal and perinatal mortality data from the local to the national level. M/PDSR can be considered as an intervention as well as an implementation process since it is a set of efforts geared towards facilitating change.[19] At all levels, the process relies on the effective reporting and assigning causes to deaths, on identifying actions that may contribute to the prevention of further deaths, assigning those actions to particular groups or individuals within a specified timeframe, and following up to ensure that those actions have been implemented. At the facility level, a six-step cycle of auditing deaths is recommended whereby: 1) cases for review are identified; 2) information on these cases is collected; 3) the information is analyzed and discussed by the MPDSR committee; 4) solutions are recommended based on the findings of the analysis; 5) solutions are implemented; and 6) feedback or reflection on if solutions were implemented and what worked or didn't in order to inform the process moving forward.[23]

In a well-functioning health system, the information from the facility-level audits feeds up into a sub-national level process whereby information about maternal and perinatal deaths is received, compiled, reviewed for completeness and any relevant actions at that level or above. The information is further analyzed and then disseminated to appropriate stakeholders, including other sub-national entities who would have their own processes (e.g. District to Province). Information from the sub-national level is compiled and sent to national level whereby further synthesis and analyses are conducted. This often leads to a national annual report that is then disseminated back to subnational and facility levels.

As a concept, M/PDSR functions at multiple levels of the health system – national, subnational, facility (and for some countries community level components are included in the process). The communication system and inter-connectedness between the different levels is an important component of M/PDSR since the process is a reporting mechanism moving continuously from bottom up – facility to national – and also from top down – national to facility. For example, recommendations to the national Ministry of Health could be identified during a facility-level audit process. This information should be fed up through the system to reach the national level decision makers. Likewise, the national level needs information from the facility-level and sub-national level in order to assess the situation of maternal and perinatal mortality in the country in order to make recommendations at sub-national and facility levels.



For peer review only - http://bmjopen.bmj.com/site/about/guidelines.xhtml

## Theory-based Implementation framework

We reviewed theories, models and frameworks consolidated by others as well as M/PDSR specific literature to determine a list of possible frameworks to consider.[13 22 33 34]

Lewis' commentary on MDSR argues the importance of considering different "cultural factors" relating to M/PDSR including factors at the individual, institutional and policy levels.[22] For example, at a micro level, an individual's willingness to "self-correct" requires commitment of staff towards conducting audit themselves, to accept open discussion with peers and to take forward the actions recommended. At a meso level, proactive institutional ethos that promotes learning as a critical part of quality improvement shapes the organizational culture. An environment open to learning also requires individual responsibility and ownership of the process, whereby clinicians need to improve their practice and change their behaviour for the betterment of maternal and perinatal health. A supportive policy and political environment (macro level) would need to be in place to initiate and support implementation.

With the understanding that M/PDSR is an intervention process functioning at multiple levels of the health system, we identified five implementation frameworks for in depth review and mapped their components with each other and in relation to M/PDSR (Supplementary File 1).[35-39] Of these frameworks, we determined the Consolidated Framework for Implementation Research (CFIR) was the most relevant foundation for developing a MPDSR implementation framework because it enables understanding of different levels and different factors that influence implementation including the intervention outcome as well as the implementation process. Since not all constructs are applicable to M/PDSR implementation and some elements missing, we further built on CFIR drawing on the other frameworks and our experience of M/PDSR to develop the theory-based conceptual framework.

Table 1 presents the conceptual framework for this scoping review. It includes four domains: intervention (M/PDSR), outer setting, inner setting, and individual. The first domain is related to the characteristics of the intervention being implemented into a particular setting. The complexity of M/PDSR as a process intervention with multi-faceted components and steps meant that we did not think two separate domains for intervention and process were needed and thus were combined. As with most interventions, there will be some adaptability at each level of M/PDSR as it is implemented in different settings and at different levels. Factors within the intervention domain for M/PDSR may include the steps of the audit cycle, cost and funding for the process, perceived legitimacy of the process as resulting in change, and the perceived ability to test, adapt and implement it. The next two domains, the inner and outer setting, continuously interface and influence each other; thus the line between them is not always clear. The outer setting includes factors external to the organization that influence implementation of M/PDSR; whereas the inner setting includes factors internal to the organization. As outer setting factors influence implementation, change occurs in the inner setting. For M/PDSR implementation, the outer setting factors include policy and planning, linkages to other actors (such as professional association), pressures to implement, community links, and communication channels. For the inner setting, implementation factors include readiness to implement, the structural characteristics of the organization implementing M/PDSR, the organizational culture, and the quality of communication and relationships, and engagement of agents of change (also called champions in some settings). The last domain considers the characteristics of the individuals involved in implementation. Factors include their individual capacity and knowledge, their motivations and commitments to the implement M/PDSR, as well as their commitment to the team or organization, and their willingness towards adapting to the



For peer review only - http://bmjopen.bmj.com/site/about/guidelines.xhtml

intervention. The specific factors considered within each domain also vary depending on the level of implementation and on the context of the implementation effort.

**Table 1: Conceptual implementation framework for M/PDSR**

| **Domain 1: Intervention/ MPDSR** |
|---|
| SERVICE DELIVERY LENS (tangible inputs) |
| Executing audit: steps of cycle implemented |
| Cost and funding for the audit process including collecting data, meeting related costs including transport, specific training, running secretariat, time |
| SOCIETAL LENS (social understanding and relationships) |
| Intervention source: Legitimacy depending on whether intervention is externally or internally developed |
| Evidence strength & quality: Evidence supporting the belief that the intervention will have desired outcomes (reduced mortality; changes undertaken to improve quality of care / "response") |
| Relative advantage: Perception of the advantage of implementing the intervention versus an alternative solution |
| SYSTEMS LENS (change dynamics) |
| Trialability: Ability to test/ pilot the intervention on a small scale, learn and revise if warranted |
| Reflectivity: feedback about the progress and quality of implementation accompanied with regular personal and team debriefing about progress and experience. |
| Adaptability: Degree to which an intervention can be tailored to meet the needs of an organization (core vs. peripheral elements) |
| Complexity: Perceived difficulty of implementation by the implementers (extent of disruption, number of elements/steps, extent of discretion, health system levels, actors) |
| **Domain 2: Outer Setting/ Broader context** |
| SERVICE DELIVERY LENS (tangible inputs) |
| Policy and planning: MPDSR policy and guidelines, Death notification requirements (legal framework for notifying deaths), Legal mandate, litigation/legal protection |
| Resource flows: any mention of funding support or resources for MPDSR (e.g. sponsors, related costs being funded/budgeted) |
| SOCIETAL LENS (social understanding and relationships) |
| Linkages to other actors: Local party, Union affiliations, Professional associations, Community organisations |
| Pressure: to implement from actors and other implementers |
| Community links: Awareness of MPDSR in the community (grassroots); community or CHW engagement and participation in MPDSR |
| SYSTEMS LENS (change dynamics) |
| Cosmopolitanism: Level of connectedness and networks with other health system levels, organizations and therefore openness or resistance to change |
| **Domain 3: Inner Setting** |
| SERVICE DELIVERY LENS (tangible inputs) |
| Readiness to implement: committees formed, training, focal point identified, availability of tools, leadership engagement and management capacity, HRH workload, access to resources |
| Structural characteristics of social architecture (characteristics of the team e.g. size, interdisciplinary nature, membership regulation) |
| Incentives/rewards (Disincentives/sanctions): Extrinsic incentives such as goal-sharing awards, performance reviews/promotions, training, tea or the consequences |



For peer review only - http://bmjopen.bmj.com/site/about/guidelines.xhtml

| |
|---|
| SOCIETAL LENS (social understanding and relationships) |
| Networks & communication: nature and quality of communication within audit team (including hierarchies, mentorship, teamwork) |
| Culture: Norms and values, organizational assumptions (blame culture vs. trust) |
| SYSTEMS LENS (change dynamics) |
| Implementation climate: explanation of environment e.g. learning climate, relative priority, if there are things mentioned that are tensions/triggers for change |
| Agents of change: Individuals who have formal or informal influence on the attitudes and beliefs of their colleagues with respect to implementing the intervention or on the implementation process overall |
| **Domain 4: Individuals** |
| SERVICE DELIVERY LENS (tangible inputs) |
| Technical skills & Knowledge: Individual staff knowledge and competencies |
| SOCIETAL LENS (social understanding and relationships) |
| Individual motivation, Self-efficacy: An individual's confidence in their capabilities to execute the implementation; individuals who are motivated |
| Individual commitment/ownership to team and organization: Individuals' perception of their commitment to the organization and their relationship |
| Individual commitment/ownership of intervention: Individuals' perception of their commitment to the intervention |
| Individual orientation: Personal traits such as tolerance of ambiguity, team player, flexibility, problem solving, critical thinking |
| SYSTEMS LENS (change dynamics) |
| Individual state of change: Phase an individual is in as he or she progresses toward skilled, enthusiastic, and sustained use of the intervention |

The final component of the theoretical conceptual framework considers three different framings or lenses through which to understand and measure health system drivers of women's and children's health.[40] A service delivery lens includes the tangible inputs needed for M/PDSR implementation; a societal lens includes constructs that focus on social understanding and relationships; and a systems lens includes constructs that emphasize change dynamics which entails adaptive learning to contexts in ways that are not always anticipated. These three lenses have been presented by George et al. (2019) as a way to describe both the tangible and intangible health systems drivers.[40] For each domain, we have categorized the constructs by these lenses.

We will test the framework on up to five different types of papers identified during the screening process and consider if any revisions need to be made. We will also undertake a consultation with experts in M/PDSR and implementation research to acquire their feedback and consider their recommendations for inclusion.

Figure 1 visualizes application of the proposed conceptual framework to the concept of M/PDSR. As an intervention, M/PDSR is presented by the grey arrows encompassing various health systems levels and implementation factors that interact dynamically. Within a country, we acknowledge the multiple health system levels – national, sub-national, facility – through which M/PDSR is operating. Within each level, there is a process for assessing the information relating to maternal and perinatal deaths (shown by the continuous circle). At each health systems level, there are different types of factors influencing implementation - outer, inner or individual. Finally, there are multiple lenses from which to understand



For peer review only - http://bmjopen.bmj.com/site/about/guidelines.xhtml

and assess implementation (service delivery, societal and systems). For the scoping review, we will extract data with consideration of these multiple levels and factors.

## Scoping review Protocol design

A scoping review was selected given the need for flexibility to explore different types of studies; and because it will facilitate a mapping and synthesis of available literature assessing implementation of M/PDSR and factors influencing implementation. [41]

The design will be guided by methods developed by Arksey and O'Malley[42] and expanded by Lavac et al. [43] with guidance from the Joanna Briggs Institute (JBI) on conducting scoping reviews.[44] Details for the proposed six stages of a scoping review are described.

## Stage 1: Identifying the research question

Our main research question is: "What do we know about implementation of maternal death audit, perinatal death audit or combined audit approaches and the factors that either support or hinder the implementation process?" We also seek to answer: "How can a theory-based conceptual implementation framework help to explain the various influencing implementation factors and their interactions?"

## Stage 2: Identify relevant studies

### *Process of search strategy*

The study will consider all literature that reports on implementation of maternal and/or perinatal death audit published in English between 2004 and July 2018 from low and middle-income countries. The start year is selected to coincide with the first World Health Organization maternal death review guideline.[27]

We will include both quantitative and qualitative research studies. Peer-review publications will be primary sources but other published and unpublished (grey) literature such as reviews, reports, and commentaries will be also taken into consideration.

An initial limited search of three online databases, which are relevant to our topic, will be undertaken using Google Scholar and PubMed to pilot the search strategy terms. Medical subject heading terms from PubMed will be used at the start to determine the words used to search in PubMed. We will combine search terms focused on maternal mortality, perinatal mortality, audit/review systems, and attributes of audit/review systems (search strategy found in Supplementary File 2).

After the initial search, we will analyze the text words contained in the title and abstract of retrieved articles, and of the index terms used to describe the articles. Revisions of our search strategy will be considered based on the findings of the initial search and incorporate additional keywords, sources and search terms as appropriate. A second search using all the identified keywords and the index terms specific to each database will be undertaken across accessible databases and websites. The search will then be performed using the following additional electronic databases and online search engines: PubMed, CINAHL, SCOPUS, Web of Science, JSTOR, LILACS, the WHO Library, Maternal Death Surveillance and Response Action Network, and Google.



For peer review only - http://bmjopen.bmj.com/site/about/guidelines.xhtml

The reference lists of all identified reports and articles will be searched for additional studies. All identified studies will be added into EndNote software and duplicate citations will be removed. We may contact the authors of primary studies or reviews for further information if necessary to provide clarity or to access additional information.

Finally, we will consult with experts in the field, including members of the World Health Organization's MPDSR Technical Working Group, to ensure we have identified all relevant literature (published and grey).

### *Characteristics of criteria*

Table 2 provides the inclusion criteria for this scoping review. For the concept component, we will only include literature that focuses on maternal or perinatal death reviews; thus excluding verbal autopsy or community death reviews, near-miss reviews, or confidential enquiries into maternal deaths. We also will exclude literature that does not specifically describe influencers of M/PDSR implementation. For example, some studies focus on the results of the audit data such as assessing cases of pre-eclampsia. If the article does not include factors exploring the implementation process, it will not be included.

**Table 2: Inclusion criteria for the scoping review**

| Components | Application to this scoping review |
|---|---|
| Concept component | • All forms of maternal and perinatal death review including obstetric audit, MPDSR, MDSR, MDR<br>• Limited to studies or perspectives that identify factors that influence the implementation process |
| Context component | • Limited to low and middle income countries listed by the World Bank in 2018. |

## Stage 3: Study selection

### *Process of screening and data extraction*

Two reviewers will independently screen titles and then abstracts to check for relevance to the review. The reviewers will regularly meet during the screening process to discuss their selection of articles and to refine screening, if needed. In the cases where abstracts are not available, the full text will be screened. All discrepancies between reviewers will be resolved by a third party.

Using the same process, the same reviewers will subsequently screen remaining full texts. All discrepancies between reviewers will be resolved by an independent third party.

## Stage 4: Data collection

A data collection instrument will be developed by the research team according to the JBI guidance. The extracted data will include study characteristics (e.g., type of reference such as article, report, study population, setting, study time period, study objective, study design). We will also consider the level or cultural factors addressed i.e. policies, law and rhetoric (macro level), internalised routine practice at the sub-national and facility levels (meso level), and individual behaviour change (micro level).[22]



For peer review only - http://bmjopen.bmj.com/site/about/guidelines.xhtml

For peer review only

For describing implementation of M/PDSR, extracted data will be based on the constructs identified in the conceptual framework. The draft data extraction components are provided in Supplementary File 3. We will pilot test the data extraction tool during a workshop and agree on any revisions. The same team members who will undertake the screening process will extract data from the selected articles. The team will engage in weekly meetings to discuss any issues or questions relating to the extraction process; decisions on extraction process will be documented. The charting table may be updated if other additional unforeseen data are identified as extraction moves forward.

## Stage 5: Data summary and synthesis of results

The review decision process will be reported using an adapted "Preferred Reporting Items for Systematic Reviews and Meta-Analyses (PRISMA) flow diagram. Data analysis will involve qualitative thematic and content analysis linked to the conceptual framework.[45]

## Stage 6: Consultation

Stakeholders will be engaged throughout the scoping review from helping to identify literature, to providing input on the conceptual framework and reviewing the findings to support interpretation. Consultations will be targeted at experts serving on the World Health Organization's MPDSR Technical Working Group, as well as the Countdown to 2030 Drivers Technical Working Group. Other experts will be identified through a snowballing approach.

## DISSEMINATION AND ETHICS

This study will contribute to the work of the Countdown to 2030 Drivers Group, which explores key contextual drivers for equitable and effective coverage of maternal and child health interventions as well as the MPDSR Technical Working Group co-led by UNFPA, UNICEF and the World Health Organization, which is tasked with advancing implementation of the intervention. This scoping review seeks to contribute specifically to the understanding how implementation of M/PDSR can drive quality improvements in service delivery responses to women and children's health. Ethics approval is not required since the scoping review methodology consists of reviewing and collecting data from publicly available sources. Consultation with key stakeholder groups will further guide dissemination efforts.

For peer review only



For peer review only - http://bmjopen.bmj.com/site/about/guidelines.xhtml

## Contributors

MK conceived of the idea, developed the research question and study methods and contributed meaningfully to the drafting and editing; she also approved the final manuscript. AG, DRW, PW, PW aided in developing the research question and study methods, contributed meaningfully to the drafting and editing, and approved the final manuscript.

## Funding

Asha George and Mary Kinney are supported by the South African Research Chair's Initiative of the Department of Science and Technology and National Research Foundation of South Africa (Grant No 82769), the South African Medical Research Council and the Countdown 2030 project funded by the Bill and Melinda Gates Foundation. Any opinion, finding and conclusion or recommendation expressed in this material is that of the author and funders do not accept any liability in this regard.

## Competing interests

None declared.

## Provenance and peer review

Not commissioned; externally peer reviewed.

## Data sharing statement

No additional data are available.

For peer review only



For peer review only - http://bmjopen.bmj.com/site/about/guidelines.xhtml

**REFERENCES**


1. Bhutta ZA, Salam RA, Lassi ZS, et al. Approaches to improve quality of care (QoC) for women and newborns: conclusions, evidence gaps and research priorities. *Reprod Health* 2014;11 Suppl 2:S5. doi: 10.1186/1742-4755-11-S2-S5
2. WHO. Standards for improving quality of maternal and newborn care in health facilities. Geneva: World Health Organization, 2016.
3. WHO. Quality, Equity, Dignity: A network for improving quality of care for maternal, newborn and child health 2017 [Available from: http://qualityofcarenetwork.org/ accessed February 22 2018.
4. Every Woman Every Child. The Global Strategy for Women's, Children's and Adolescents' Health. New York, NY: Every Woman Every Child, 2015.
5. WHO. Every Newborn: An action plan to end preventable newborn deaths. Geneva: World Health Organization, 2014.
6. WHO. Strategies toward ending preventable maternal mortality (EPMM). Geneva: World Health Organization, 2015.
7. Kruk ME, Pate M, Mullan Z. Introducing The Lancet Global Health Commission on High-Quality Health Systems in the SDG Era. *The Lancet Global health* 2017;5(5):e480-e81. doi: 10.1016/S2214-109X(17)30101-8
8. Ivers N, Jamtvedt G, Flottorp S, et al. Audit and feedback: effects on professional practice and healthcare outcomes. *Cochrane Database Syst Rev* 2012;6:CD000259. doi: 10.1002/14651858.CD000259.pub3
9. Akachi Y, Kruk ME. Quality of care: measuring a neglected driver of improved health. *Bull World Health Organ* 2017;95(6):465-72. doi: 10.2471/BLT.16.180190
10. Heiby JR, Armbruster D, Jacobs TA. Better care for every patient, every time: improving quality in low health systems. *Bjog* 2014;121 Suppl 4:4-7. doi: 10.1111/1471-0528.12903
11. Langley A, Denis JL. Beyond evidence: the micropolitics of improvement. *BMJ quality & safety* 2011;20 Suppl 1:i43-6. doi: 10.1136/bmjqs.2010.046482
12. Davidoff F, Dixon-Woods M, Leviton L, et al. Demystifying theory and its use in improvement. *BMJ quality & safety* 2015;24(3):228-38. doi: 10.1136/bmjqs-2014-003627
13. Nilsen P. Making sense of implementation theories, models and frameworks. *Implement Sci* 2015;10:53. doi: 10.1186/s13012-015-0242-0
14. Persson LA. Bridging the quality chasm in maternal, newborn, and child healthcare in low- and middle-income countries. *PLoS medicine* 2017;14(12):e1002465. doi: 10.1371/journal.pmed.1002465
15. Topp SM. The Lancet Global Health Commission on High Quality Health Systems-where's the complexity? *The Lancet Global health* 2017;5(6):e571. doi: 10.1016/S2214-109X(17)30176-6
16. Kruk ME, Marchant T, Doubova S, et al. The Lancet Global Health Commission on High Quality Health Systems-where's the complexity? - Authors' reply. *The Lancet Global health* 2017;5(6):e572. doi: 10.1016/S2214-109X(17)30178-X
17. Hulscher ME, Schouten LM, Grol RP, et al. Determinants of success of quality improvement collaboratives: what does the literature show? *BMJ quality & safety* 2013;22(1):19-31. doi: 10.1136/bmjqs-2011-000651
18. George A, Scott K, Garimella S, et al. Anchoring contextual analysis in health policy and systems research: A narrative review of contextual factors influencing health committees in low and middle income countries. *Soc Sci Med* 2015;133:159-67. doi: 10.1016/j.socscimed.2015.03.049
19. Bauer MS, Damschroder L, Hagedorn H, et al. An introduction to implementation science for the non-specialist. *BMC Psychol* 2015;3:32. doi: 10.1186/s40359-015-0089-9




For peer review only - http://bmjopen.bmj.com/site/about/guidelines.xhtml

20. Independent Expert Review Group. A post-2015 vision. The Third Report of the Independent Expert Review Group on Information and Accountability for Women's and Children's Health. Geneva: World Health Organization, 2014.
21. Hounton S, De Bernis L, Hussein J, et al. Towards elimination of maternal deaths: maternal deaths surveillance and response. *Reprod Health* 2013;10:1. doi: 10.1186/1742-4755-10-1
22. Lewis G. The cultural environment behind successful maternal death and morbidity reviews. *Bjog* 2014;121 Suppl 4:24-31. doi: 10.1111/1471-0528.12801
23. WHO. Making every baby count: audit and review of stillbirths and neonatal deaths. Geneva: World Health Organization, 2016.
24. MDSR Action Network Evidence 4 Action; 2017 [Available from: http://mdsr-action.net/ accessed 5 September 2017.
25. WHO. Global Monitoring of Implementation of Maternal Death Surveillance and Response (MDSR) 2017 [Available from: http://www.who.int/maternal_child_adolescent/epidemiology/maternal-death-surveillance/global-monitoring/en/ accessed 5 September 2017.
26. WHO. Time to respond: a report on the global implementation of maternal death surveillance and response (MDSR). Geneva: World Health Organization, 2016.
27. WHO. Beyond the numbers: Reviewing maternal deaths and complications to make pregnancy safer. Geneva: World Health Organization 2004.
28. WHO. Maternal death surveillance and response: technical guidance. Geneva: World Health Organization, 2013.
29. Kerber KJ, Mathai M, Lewis G, et al. Counting every stillbirth and neonatal death through mortality audit to improve quality of care for every pregnant woman and her baby. *BMC Pregnancy Childbirth* 2015;15 Suppl 2:S9. doi: 10.1186/1471-2393-15-S2-S9
30. Martin Hilber A, Blake C, Bohle LF, et al. Strengthening accountability for improved maternal and newborn health: A mapping of studies in Sub-Saharan Africa. *Int J Gynaecol Obstet* 2016;135(3):345-57. doi: 10.1016/j.ijgo.2016.09.008
31. Pattinson R, Kerber K, Waiswa P, et al. Perinatal mortality audit: counting, accountability, and overcoming challenges in scaling up in low- and middle-income countries. *Int J Gynaecol Obstet* 2009;107 Suppl 1:S113-21, S21-2. doi: S0020-7292(09)00362-2 [pii]

10.1016/j.ijgo.2009.07.011 [published Online First: 2009/10/10]
32. Smith H, Ameh C, Roos N, et al. Implementing maternal death surveillance and response: a review of lessons from country case studies. *BMC Pregnancy Childbirth* 2017;17(1):233. doi: 10.1186/s12884-017-1405-6
33. Nilsen P, Stahl C, Roback K, et al. Never the twain shall meet?--a comparison of implementation science and policy implementation research. *Implement Sci* 2013;8:63. doi: 10.1186/1748-5908-8-63
34. The Centre for Research in Implementation Science and Prevention (CRISP). Dissemination & implementation models in health research & practice 2015 [Available from: http://dissemination-implementation.com/index.aspx.
35. Kitson AL, Rycroft-Malone J, Harvey G, et al. Evaluating the successful implementation of evidence into practice using the PARiHS framework: theoretical and practical challenges. *Implement Sci* 2008;3:1. doi: 10.1186/1748-5908-3-1
36. Damschroder LJ, Aron DC, Keith RE, et al. Fostering implementation of health services research findings into practice: a consolidated framework for advancing implementation science. *Implement Sci* 2009;4:50. doi: 10.1186/1748-5908-4-50



For peer review only - http://bmjopen.bmj.com/site/about/guidelines.xhtml

37. Pfadenhauer LM, Gerhardus A, Mozygemba K, et al. Making sense of complexity in context and implementation: the Context and Implementation of Complex Interventions (CICI) framework. *Implement Sci* 2017;12(1):21. doi: 10.1186/s13012-017-0552-5
38. May CR, Johnson M, Finch T. Implementation, context and complexity. *Implement Sci* 2016;11(1):141. doi: 10.1186/s13012-016-0506-3
39. Chambers DA, Glasgow RE, Stange KC. The dynamic sustainability framework: addressing the paradox of sustainment amid ongoing change. *Implement Sci* 2013;8:117. doi: 10.1186/1748-5908-8-117
40. George A, al. e. Lenses and levels: the why, what and how of measuring health system drivers of women's, children's and adolescents' health with a governance focus. *BMJ global health* 2019 (in press)
41. Tricco AC, Lillie E, Zarin W, et al. A scoping review on the conduct and reporting of scoping reviews. *BMC medical research methodology* 2016;16:15. doi: 10.1186/s12874-016-0116-4
42. Arksey H, HO'Malley L. Scoping studies: towards a methodological framework. *Int J Soc Res Methodol* 2005;8(1):19-32. doi: 10.1080/1364557032000119616
43. Levac D, Colquhoun H, O'Brien KK. Scoping studies: advancing the methodology. *Implement Sci* 2010;5:69. doi: 10.1186/1748-5908-5-69
44. Joanna Briggs Institute Reviewer's Manual: 2015 edition. Adelaide, SA: Joanna Briggs Institute, 2015.
45. Vaismoradi M, Turunen H, Bondas T. Content analysis and thematic analysis: Implications for conducting a qualitative descriptive study. *Nurs Health Sci* 2013;15(3):398-405. doi: 10.1111/nhs.12048

For peer review only



For peer review only - http://bmjopen.bmj.com/site/about/guidelines.xhtml

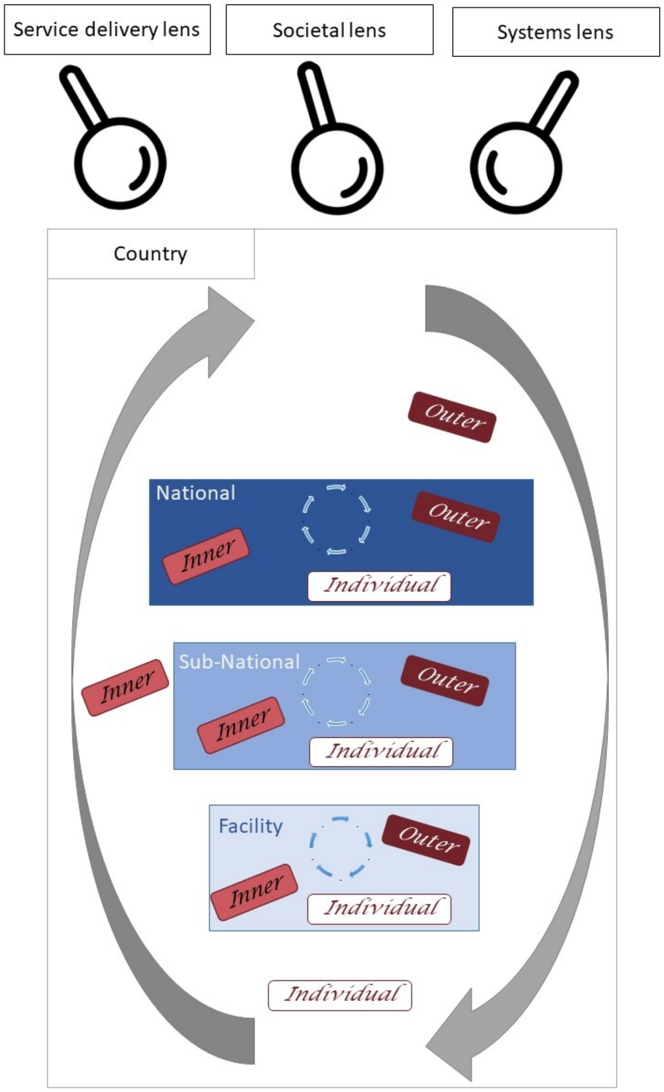


Figure 1: Conceptual framework for implementation of M/PDSR

190x338mm (96 x 96 DPI)

Supplementary Files for *Implementation of maternal and perinatal death reviews: a scoping review*

**SUPPLEMENTARY FILE 1**

**Table S1.1: Frameworks considered when developing the conceptual frameworks for the scoping review**

| Framework | Level | Characteristic | Pros - why we considered it | Cons – why we decided not to use it |
|---|---|---|---|---|
| Context and Implementation of Complex Intervention - CICI Framework (Pfadenhauer) [1] | Macro, Meso, Micro | Comprises three dimensions which interact: Context (i.e., geographical, epidemiological, socio-cultural, socio-economic, ethical, legal, political); implementation (i.e., implementation theory, process, strategies, agents and outcomes); setting (specific physical location). | A determinant framework that seeks to conceptualise, describe and understand the multiple influences on implementation outcomes; Enables understanding of implementation process at multiple levels across different factors; Provides data extraction tools for qualitative and quantitative reviews | It focuses on the operationalization of context, setting and implementation *not on the intervention itself.* Yet we wanted to understand more about the how the intervention is working and being implemented. |
| Dynamic sustainability framework (Chambers) [2] | Macro, Meso, | Considers the intervention, the context and the broader ecological system over time | A new paradigm to consider the long-term use and ongoing improvement of interventions; posits that ongoing QI is the aim | Does not provide enough focus on the implementation process now – rather would allow for exploring implementation of audit over time to see if process leads to change (which is not purpose of scoping review) |
| Promoting Action on Research Implementation in Health Services - PARiHS (Kitson) [3] | Meso | Considers the interactions between three key elements: Evidence, Context, Facilitation | It argues that successful implementation (SI) of evidence into practice had as much to do with the context or setting where the new evidence was being | Focuses on knowledge translation component of implementation. Many of the constructs do not relate to what we think would be needed to |



For peer review only - http://bmjopen.bmj.com/site/about/guidelines.xhtml

| | | | | |
|---|---|---|---|---|
| | | | introduced and how it was introduced | understand the MPDSR process |
| Consolidated Framework for Implementation Research - CFIR (Damschroder) [4] | Macro, Meso, Macro | Comprises five major domains and considers their interactions: the intervention, inner and outer setting, the individuals involved, and the process by which implementation is accomplished | Enables understanding of different levels and different factors that influence implementation; it includes the intervention outcome but also allows for understanding process as well as influencing factors at different levels | Not all constructs are applicable to MPDSR implementation and some elements of what we seek to understand might be missing |
| Normalization Process Theory (May) [5] | Meso, Micro | Comprises four constructs: Coherence, Cognitive Participation, Collective Action, and Reflexive Monitoring | it explores the fluid, dynamic and interactive processes between context, actors and objects Investigates the process by which an intervention becomes embedded | Does not include enough factors that should be considered for understanding implementation factors (focuses only on meso, micro level) |



For peer review only - http://bmjopen.bmj.com/site/about/guidelines.xhtml

**SUPPLEMENTARY FILE 2: SEARCH STRATEGY**

Search terms used for search engines from January 2004 to July 2018:

("maternal mortality" OR "perinatal death" OR "maternal death" OR "perinatal mortality" OR "fetal mortality" OR "stillbirth") **AND** (“audit” OR "surveillance and response")

**Table 2.1 Concept and search terms included**

| Concept | Search terms |
|---|---|
| Maternal & Perinatal death | maternal mortality, maternal death, stillbirth, fetal mortality, perinatal death, perinatal mortality |
| Audit | Audit, surveillance and response |

For peer review only

**SUPPLEMENTARY FILE 3: DRAFT DATA EXTRACTION FORM**

| | | |
|---|---|---|
| | Reviewer assigned | |
| | Inclusion | Yes<br>No |
| **Reference details** | Ref ID | |
| | Author | |
| | Title | |
| | Year | |
| | Abstract | |
| | Journal | |
| | Volume | |
| | Issue | |
| | Pages | |
| | URL | |
| | Source | Database<br>Online search<br>Consultation |
| Background to reference | Country of first affiliation | |
| | Country of Study | Name country<br>Multiple<br>N/A |
| | Country Setting | Upper MI<br>Lower MI<br>LIC<br>LMIC<br>Not specified (add to notes) |
| | Region of World | Sub-Saharan Africa<br>East Asia & Pacific<br>Latin America & Caribbean<br>South Asia<br>Europe & Central Asia<br>Middle East & North Africa<br>International |
| | Organization Type of authors | Government<br>NGO<br>University/academic institution<br>Independent<br>Mixed including government<br>Mixed not including government<br>Other (add note) |
| | Funder type (sector) | Government<br>Multilateral (e.g. WHO, World Bank, UN)<br>Bilateral (e.g. USAID, DIFD, IDRC)<br>Foundation (e.g. BMGF, Rockefeller)<br>Mixed includes government |



For peer review only - http://bmjopen.bmj.com/site/about/guidelines.xhtml

| | | |
|---|---|---|
| | | Mixed does not include government<br>No funding received |
| | Notes | |
| Content of reference | Focus of the audit | Maternal & perinatal<br>Maternal<br>Perinatal |
| | Notes | |
| | History/timeline | Yes<br>No |
| | Notes | |
| | Scale | National<br>Subnational<br>Selected Facilities<br>Combination Of Levels<br>Global<br>Multicountry<br>Other |
| | Notes | |
| Reference type | Document type | Academic journal article<br>Academic journal commentary<br>Academic review<br>Grey lit online<br>Grey lit not online<br>other |
| | Methods section | Yes<br>No |
| | Research Design | Quantitative<br>Qualitative<br>Mixed<br>NA |
| | Level of Study | Macro<br>Meso<br>Micro<br>Combination |
| | Notes | |
| Domain 1: Intervention/MPDSR | Executing audit | Described<br>Not described |
| | Notes | |
| | Cost & funding | Described<br>Not described |
| | Notes | |
| | Intervention source | Described<br>Not described |
| | Notes | |
| | Evidence strength & quality | Described<br>Not described |



For peer review only - http://bmjopen.bmj.com/site/about/guidelines.xhtml

| | | |
|---|---|---|
| | Notes | |
| | Relative advantage | Described<br>Not described |
| | Notes | |
| | Trialability | Described<br>Not described |
| | Notes | |
| | Reflectivity | Described<br>Not described |
| | Notes | |
| | Adaptability | Described<br>Not described |
| | Notes | |
| | Complexity | Described<br>Not described |
| | Notes | |
| Domain 2: Outer Setting | Policy and planning | Described<br>Not described |
| | Notes | |
| | Resource flows | Described<br>Not described |
| | Notes | |
| | Linkages to other actors | Described<br>Not described |
| | Notes | |
| | Pressure | Described<br>Not described |
| | Notes | |
| | Community links | Described<br>Not described |
| | Notes | |
| | Cosmopolitanism | Described<br>Not described |
| | Notes | |
| Domain 3: Inner Setting | Readiness to implement | Described<br>Not described |
| | Notes | |
| | Structural characteristics of social architecture | Described<br>Not described |
| | Notes | |
| | Incentive/rewards | Described<br>Not described |
| | Notes | |
| | Networks & communication | Described<br>Not described |

| | | |
|---|---|---|
| | Notes | |
| | Culture | Described<br>Not described |
| | Notes | |
| | Implementation climate | Described<br>Not described |
| | Notes | |
| | Agents of change | Described<br>Not described |
| | Notes | |
| Domain 4: Individuals | Tech skills & knowledge | Described<br>Not described |
| | Notes | |
| | Individual motivation, Self-efficacy | Described<br>Not described |
| | Notes | |
| | Individual commitment | Described<br>Not described |
| | Notes | |
| | Individual orientation | Described<br>Not described |
| | Notes | |
| | Individual state of change | Described<br>Not described |
| | Notes | |

**REFERENCES**


1. Pfadenhauer LM, Gerhardus A, Mozygemba K, Lysdahl KB, Booth A, Hofmann B et al. Making sense of complexity in context and implementation: the Context and Implementation of Complex Interventions (CICI) framework. Implement Sci. 2017; 12(1):21.
2. Chambers DA, Glasgow RE,Stange KC. The dynamic sustainability framework: addressing the paradox of sustainment amid ongoing change. Implement Sci. 2013; 8:117.
3. Kitson AL, Rycroft-Malone J, Harvey G, McCormack B, Seers K,Titchen A. Evaluating the successful implementation of evidence into practice using the PARiHS framework: theoretical and practical challenges. Implement Sci. 2008; 3:1.
4. Damschroder LJ, Aron DC, Keith RE, Kirsh SR, Alexander JA,Lowery JC. Fostering implementation of health services research findings into practice: a consolidated framework for advancing implementation science. Implement Sci. 2009; 4:50.
5. Murray E, Treweek S, Pope C, MacFarlane A, Ballini L, Dowrick C et al. Normalisation process theory: a framework for developing, evaluating and implementing complex interventions. BMC medicine. 2010; 8:63.




For peer review only - http://bmjopen.bmj.com/site/about/guidelines.xhtml

BMJ Open

# Implementation of maternal and perinatal death reviews: a scoping review protocol

| | |
|---|---|
| Journal: | *BMJ Open* |
| Manuscript ID | bmjopen-2019-031328.R1 |
| Article Type: | Protocol |
| Date Submitted by the Author: | 09-Oct-2019 |
| Complete List of Authors: | Kinney, Mary; University of the Western Cape Faculty of Community and Health Sciences, School of Public Health<br>Walugembe, David; University of Western Ontario, School of Health Studies and Faculty of Information and Media Studies<br>Wanduru, Phillip; Makerere University<br>Waiswa, Peter; Makerere University College of Health Sciences, Schollo of Public health; Karolinska Institutet,<br>George, Asha S. ; University of the Western Cape, |
| <b>Primary Subject Heading</b>: | Public health |
| Secondary Subject Heading: | Obstetrics and gynaecology, Paediatrics, Health policy |
| Keywords: | Maternal medicine < OBSTETRICS, Clinical audit < HEALTH SERVICES ADMINISTRATION & MANAGEMENT, Surveillance, implementation, Quality in health care < HEALTH SERVICES ADMINISTRATION & MANAGEMENT, perinatal |
| | |

SCHOLARONE™
Manuscripts

**Title**: Implementation of maternal and perinatal death reviews: a scoping review protocol

**Authors**: Mary V. Kinney*, David Roger Walugembe, Phillip Wanduru, Peter Waiswa, Asha George

Affiliations:

- Mary V. Kinney, School of Public Health, University of the Western Cape, Private Bag X17, Bellville 7535 South Africa; email: mkinney@uwc.ac.za; telephone: (+27) 834448211
- David Roger Walugembe, School of Health Studies and Faculty of Information and Media Studies, The University of Western Ontario, London, ON, Canada
- Phillip Wanduru, College of Health Sciences, Makerere University, Kampala, Uganda
- Peter Waiswa, College of Health Sciences, Makerere University School of Public Health, Kampala, Uganda
- Asha S. George, School of Public Health, University of the Western Cape, Bellville, South Africa

* Corresponding author

For peer review only

## ABSTRACT

### Introduction

Maternal and perinatal death surveillance and response (MPDSR), or any related form of audit, is a systematic process used to prevent future maternal and perinatal deaths. While the existence of MPDSR policies is routinely measured, measurement and understanding of policy implementation has lagged behind. In this paper, we present a theory-based conceptual framework for understanding MPDSR implementation as well as a scoping review protocol to understand factors influencing MPDSR implementation in low-and middle-income countries (LMICs).

### Methods and analysis

The Consolidated Framework for Implementation Research will inform the development of a theory-based conceptual framework for MPDSR implementation. The methodology for the scoping review will be guided by an adapted Arksey and O'Malley approach. Documents will include published and grey literature sourced from electronic databases (PubMed, CINAHL, SCOPUS, Web of Science, JSTOR, LILACS), the WHO Library, Maternal Death Surveillance and Response Action Network, Google, the reference lists of key studies, and key experts. Two reviewers will independently screen titles, abstracts and full studies for inclusion. All discrepancies will be resolved by an independent third party. We will include studies published in English from 2004 to July 2018 that present results on factors influencing implementation of MPDSR, or any related form. Qualitative content and thematic analysis will be applied to extracted data according to the theory-based conceptual framework. Stakeholders will be consulted at various stages of the process.

### Dissemination and Ethics

The scoping review will synthesise implementation factors relating to MPDSR in LMIC as described in the literature. This review will contribute to the work of the Countdown to 2030 Drivers Group, which seeks to explore key contextual drivers for equitable and effective coverage of maternal and child health interventions. Ethics approval is not required. The results will be disseminated through various channels, including a peer-reviewed publication.


## STRENGTHS AND LIMITATIONS OF THE STUDY

- This will be the first scoping review to identify and describe implementation factors relating to maternal and perinatal death surveillance and response (MPDSR) in low and middle-income countries.
- The search strategy includes six electronic databases with peer-reviewed literature as well as three online search engines to identify published and grey literature including academic research articles, commentaries, other related reviews, and reports.
- The results will be presented using an adapted theory-based conceptual framework for MPDSR implementation, and data analysis will involve qualitative thematic and content analysis linked to this framework.
- Stakeholders including the World Health Organization's MPDSR Technical Working Group as well as the Countdown to 2030 Drivers Technical Working Group will be consulted and engaged throughout the study review process.



For peer review only

For peer review only - http://bmjopen.bmj.com/site/about/guidelines.xhtml

- Some literature or components of the identified literature may not be included in the results of this scoping review due to the limitations of the search criteria, notably around language (English only) and time span (from 2004-July 2018) and the search process (e.g. not all grey literature can be identified).

For peer review only



For peer review only - http://bmjopen.bmj.com/site/about/guidelines.xhtml

## INTRODUCTION

Most deliveries in developing countries now happen in hospitals and clinics making facility-based maternal and newborn care a global health imperative for achieving the Sustainable Development Goal for health.[1 2] Maternal death surveillance and response (MDSR), perinatal death audit, or a joint maternal and perinatal death surveillance and response (MPDSR) is one process used to prevent maternal and perinatal deaths.[3-5] Maternal and/or perinatal death surveillance and response, referred to in this paper as M/PDSR, is an established mechanism to examine the circumstances surrounding each death to prevent future deaths.[6] It requires continuous application of monitoring-review-act cycles[7] to capture information on the number and causes of deaths, with systematic, critical analysis of the care received for a sample of or  for all cases, in a no-blame, interdisciplinary setting, with a view to improving the care provided to all mothers and babies.[8]  The potential for MPDSR to improve mortality outcomes only occurs if the audit cycle is completed and implemented overtime triggering iterative cycles of improvement.[9-11]

In the past fifteen years, there has been momentum to strengthen clinical audit practice for maternal and perinatal death,[12-15] including the development of global technical guidelines.[8 16] Many low- and middle-income countries (LMIC) have adopted national guidelines, however few have robust MPDSR systems.[9]  A growing number of studies have investigated the implementation of M/PDSR in selected countries, and some reviews have explored implementation factors for maternal death reviews or perinatal death audits, separately. For example, a structured literature review of accountability mechanisms for maternal and newborn health in sub-Saharan Africa found MDSR the most common mechanism for performance accountability.[17] A systematic review of facility-based perinatal mortality audit in LMIC countries in 2009 identified ten low-quality evaluations with mortality outcome data.[10] A literature review conducted in 2015 on facility based perinatal audits explored enablers and barriers according to the health system building blocks.[9]

While valuable contributions to the literature, these previous reviews did not considered implementation theory to assess implementation of M/PDSR nor of the full range of types of maternal and/or perinatal death reviews.[9 17 18] Implementation theory allows for more complex interventions to be unpacked and examined.[19-24] This approach enables exploration of issues, such as trust, credibility, relationships and hierarchies to understand factors that support or hinder implementation.[25] Interventions seeking to improve facility-based care are often ongoing processes that are complex, fluid and context specific.[7 19 24 26]  A variety of factors,[19-24 27] including context,[28] can influence implementation of these types of interventions. With rising attention on facility-based maternal and newborn health care,[5 29-31] more needs to be understood about the implementation of M/PDSR.

### Study rationale

Global agencies, such as the World Health Organization, have created guidelines for M/PDSR and are encouraging LMIC to move forward with implementation.[8 14] Further understanding of the enablers and barriers of implementation in LMIC is needed to support roll out of this intervention across and within countries. A rigorous, scoping review has not yet been undertaken to map publications in LMIC on factors influencing implementation of M/PDSR in ways that are inclusive of either maternal and/or perinatal death audits.



For peer review only - http://bmjopen.bmj.com/site/about/guidelines.xhtml

## Study Objectives

To map and synthesise the available literature on the factors that support or hinder M/PDSR implementation using a theory-based conceptual implementation framework. We will also explore common, if any, implementation factors between MDSR, PDSR or MPDSR.

# METHODS AND ANALYSIS

## Conceptual model

This section of the protocol presents a proposed theory-based conceptual framework, which will be pilot tested and adapted for the data extraction and analysis. To develop the framework, we considered conceptualization of the M/PDSR as an intervention process and reviewed various theory-based implementation frameworks.

### Conceptualizing M/PDSR

M/PDSR is a continuous action cycle for quality improvement that links maternal and perinatal mortality data from the local to the national level. M/PDSR can be considered as an intervention as well as an implementation process since it is a set of efforts geared towards facilitating change.[25] At all levels, the process relies on the effective reporting and assigning causes to deaths, on identifying actions that may contribute to the prevention of further deaths, assigning those actions to particular groups or individuals within a specified timeframe, and following up to ensure that those actions have been implemented. At the facility level, a six-step cycle of auditing deaths is recommended whereby: 1) cases for review are identified; 2) information on these cases is collected; 3) the information is analyzed and discussed by the MPDSR committee; 4) solutions are recommended based on the findings of the analysis; 5) solutions are implemented; and 6) feedback or reflection on if solutions were implemented and what worked or didn't in order to inform the process moving forward.[8]

In a well-functioning health system, the information from the facility-level audits feeds up into a sub-national level process whereby information about maternal and perinatal deaths is received, compiled, reviewed for completeness and any relevant actions at that level or above. The information is further analyzed and then disseminated to appropriate stakeholders, including other sub-national entities who would have their own processes (e.g. District to Province). Information from the sub-national level is compiled and sent to national level whereby further synthesis and analyses are conducted. This often leads to a national annual report that is then disseminated back to subnational and facility levels.

As a concept, M/PDSR functions at multiple levels of the health system – national, subnational, facility (and for some countries community level components are included in the process). The communication system and inter-connectedness between the different levels is an important component of M/PDSR since the process is a reporting mechanism moving continuously from bottom up – facility to national – and also from top down – national to facility. For example, recommendations to the national Ministry of Health could be identified during a facility-level audit process. This information should be fed up through the system to reach the national level decision makers. Likewise, the national level needs information from the facility-level and sub-national level in order to assess the situation of maternal and perinatal mortality in the country in order to make recommendations at sub-national and facility levels.



For peer review only - http://bmjopen.bmj.com/site/about/guidelines.xhtml

## Theory-based Implementation framework

We reviewed theories, models and frameworks consolidated by others as well as M/PDSR specific literature to determine a list of possible frameworks to consider.[6 27 32 33]

Lewis' commentary on MDSR argues the importance of considering different "cultural factors" relating to M/PDSR including factors at the individual, institutional and policy levels.[6] For example, at a micro level, an individual's willingness to "self-correct" requires commitment of staff towards conducting audit themselves, to accept open discussion with peers and to take forward the actions recommended. At a meso level, proactive institutional ethos that promotes learning as a critical part of quality improvement shapes the organizational culture. An environment open to learning also requires individual responsibility and ownership of the process, whereby clinicians need to improve their practice and change their behaviour for the betterment of maternal and perinatal health. A supportive policy and political environment (macro level) would need to be in place to initiate and support implementation.[6]

With the understanding that M/PDSR is an intervention process functioning at multiple levels of the health system, we identified five implementation frameworks for in depth review and mapped their components with each other and in relation to M/PDSR (Supplementary File 1).[34-38] Our mapping process found that both the Context and Implementation of Complex Intervention Framework[36] and the Dynamic Sustainability Framework[38] did not provide enough consideration to the implementation process of the intervention. The Promoting Action on Research Implementation in Health Services Framework[34] and the Normalization Process Theory[39] had strengths especially at the meso and micro level for understanding implementation processes; however there was not enough overlap with the concepts identified for M/PDSR implementation. The Consolidated Framework for Implementation Research (CFIR) was found to be the most relevant foundation for developing a MPDSR implementation framework because it enables understanding of different levels and different factors that influence implementation including the intervention outcome as well as the implementation process.[35] Since not all constructs are applicable to M/PDSR implementation and some elements missing, we further built on CFIR drawing on the other frameworks and our experience of M/PDSR to develop the theory-based conceptual framework.

Table 1 presents the conceptual framework for this scoping review. It includes four domains: intervention (M/PDSR), outer setting, inner setting, and individual. The first domain is related to the characteristics of the intervention being implemented into a particular setting. The complexity of M/PDSR as a process intervention with multi-faceted components and steps meant that we did not think two separate domains for intervention and process were needed and thus were combined. As with most interventions, there will be some adaptability at each level of M/PDSR as it is implemented in different settings and at different levels. Factors within the intervention domain for M/PDSR may include the steps of the audit cycle, cost and funding for the process, perceived legitimacy of the process as resulting in change, and the perceived ability to test, adapt and implement it. The next two domains, the inner and outer setting, continuously interface and influence each other; thus the line between them is not always clear. The outer setting includes factors external to the organization that influence implementation of M/PDSR; whereas the inner setting includes factors internal to the organization. As outer setting factors influence implementation, change occurs in the inner setting. For M/PDSR implementation, the outer setting factors include policy and planning, linkages to other actors (such as professional association), pressures to implement, community links, and communication channels. For


For peer review only - http://bmjopen.bmj.com/site/about/guidelines.xhtml

the inner setting, implementation factors include readiness to implement, the structural characteristics of the organization implementing M/PDSR, the organizational culture, and the quality of communication and relationships, and engagement of agents of change (also called champions in some settings). The last domain considers the characteristics of the individuals involved in implementation. Factors include their individual capacity and knowledge, their motivations and commitments to the implement M/PDSR, as well as their commitment to the team or organization, and their willingness towards adapting to the intervention. The specific factors considered within each domain also vary depending on the level of implementation and on the context of the implementation effort.

**Table 1: Conceptual implementation framework for M/PDSR**

| **Domain 1: Intervention/ MPDSR** |
|---|
| SERVICE DELIVERY LENS (tangible inputs) |
| Executing audit: steps of cycle implemented |
| Cost and funding for the audit process including collecting data, meeting related costs including transport, specific training, running secretariat, time |
| SOCIETAL LENS (social understanding and relationships) |
| Intervention source: Legitimacy depending on whether intervention is externally or internally developed |
| Evidence strength & quality: Evidence supporting the belief that the intervention will have desired outcomes (reduced mortality; changes undertaken to improve quality of care / "response") |
| Relative advantage: Perception of the advantage of implementing the intervention versus an alternative solution |
| SYSTEMS LENS (change dynamics) |
| Trialability: Ability to test/ pilot the intervention on a small scale, learn and revise if warranted |
| Reflectivity: feedback about the progress and quality of implementation accompanied with regular personal and team debriefing about progress and experience. |
| Adaptability: Degree to which an intervention can be tailored to meet the needs of an organization (core vs. peripheral elements) |
| Complexity: Perceived difficulty of implementation by the implementers (extent of disruption, number of elements/steps, extent of discretion, health system levels, actors) |
| **Domain 2: Outer Setting/ Broader context** |
| SERVICE DELIVERY LENS (tangible inputs) |
| Policy and planning: MPDSR policy and guidelines, Death notification requirements (legal framework for notifying deaths), Legal mandate, litigation/legal protection |
| Resource flows: any mention of funding support or resources for MPDSR (e.g. sponsors, related costs being funded/budgeted) |
| SOCIETAL LENS (social understanding and relationships) |
| Linkages to other actors: Local party, Union affiliations, Professional associations, Community organisations |
| Pressure: to implement from actors and other implementers |
| Community links: Awareness of MPDSR in the community (grassroots); community or CHW engagement and participation in MPDSR |
| SYSTEMS LENS (change dynamics) |
| Cosmopolitanism: Level of connectedness and networks with other health system levels, organizations and therefore openness or resistance to change |
| **Domain 3: Inner Setting** |
| SERVICE DELIVERY LENS (tangible inputs) |



For peer review only - http://bmjopen.bmj.com/site/about/guidelines.xhtml

| |
|---|
| Readiness to implement: committees formed, training, focal point identified, availability of tools, leadership engagement and management capacity, HRH workload, access to resources |
| Structural characteristics of social architecture (characteristics of the team e.g. size, interdisciplinary nature, membership regulation) |
| Incentives/rewards (Disincentives/sanctions): Extrinsic incentives such as goal-sharing awards, performance reviews/promotions, training, tea or the consequences |
| SOCIETAL LENS (social understanding and relationships) |
| Networks & communication: nature and quality of communication within audit team (including hierarchies, mentorship, teamwork) |
| Culture: Norms and values, organizational assumptions (blame culture vs. trust) |
| SYSTEMS LENS (change dynamics) |
| Implementation climate: explanation of environment e.g. learning climate, relative priority, if there are things mentioned that are tensions/triggers for change |
| Agents of change: Individuals who have formal or informal influence on the attitudes and beliefs of their colleagues with respect to implementing the intervention or on the implementation process overall |
| **Domain 4: Individuals** |
| SERVICE DELIVERY LENS (tangible inputs) |
| Technical skills & Knowledge: Individual staff knowledge and competencies |
| SOCIETAL LENS (social understanding and relationships) |
| Individual motivation, Self-efficacy: An individual's confidence in their capabilities to execute the implementation; individuals who are motivated |
| Individual commitment/ownership to team and organization: Individuals' perception of their commitment to the organization and their relationship |
| Individual commitment/ownership of intervention: Individuals' perception of their commitment to the intervention |
| Individual orientation: Personal traits such as tolerance of ambiguity, team player, flexibility, problem solving, critical thinking |
| SYSTEMS LENS (change dynamics) |
| Individual state of change: Phase an individual is in as he or she progresses toward skilled, enthusiastic, and sustained use of the intervention |

The final component of the theoretical conceptual framework considers three different framings or lenses through which to understand and measure health system drivers of women's and children's health.[40] A service delivery lens includes the tangible inputs needed for M/PDSR implementation; a societal lens includes constructs that focus on social understanding and relationships; and a systems lens includes constructs that emphasise change dynamics which entails adaptive learning to contexts in ways that are not always anticipated. These three lenses have been presented by George et al. (2019) as a way to describe both the tangible and intangible health systems drivers.[40] For each domain, we have categorised the constructs by these lenses.

We will test the framework on up to five different types of papers identified during the screening process and consider if any revisions need to be made. We will also undertake a consultation with experts in M/PDSR and implementation research to acquire their feedback and consider their recommendations for inclusion.



For peer review only - http://bmjopen.bmj.com/site/about/guidelines.xhtml

Figure 1 visualises application of the proposed conceptual framework to the concept of M/PDSR. As an intervention, M/PDSR is presented by the grey arrows encompassing various health systems levels and implementation factors that interact dynamically. Within a country, we acknowledge the multiple health system levels – national, sub-national, facility – through which M/PDSR is operating. Within each level, there is a process for assessing the information relating to maternal and perinatal deaths (shown by the continuous circle). At each health systems level, there are different types of factors influencing implementation - outer, inner or individual. Finally, there are multiple lenses from which to understand and assess implementation (service delivery, societal and systems). For the scoping review, we will extract data with consideration of these multiple levels and factors.

## Scoping review Protocol design

A scoping review was selected given the need for flexibility to explore different types of studies; and because it will facilitate a mapping and synthesis of available literature assessing implementation of M/PDSR and factors influencing implementation. [41]

The design will be guided by methods developed by Arksey and O'Malley[42] and expanded by Lavac et al. [43] with guidance from the Joanna Briggs Institute (JBI) on conducting scoping reviews.[44] Details for the proposed six stages of a scoping review are described.

## Stage 1: Identifying the research question

Our main research question is: "What do we know about implementation of maternal death audit, perinatal death audit or combined audit approaches and the factors that either support or hinder the implementation process?" We also seek to answer: "How can a theory-based conceptual implementation framework help to explain the various influencing implementation factors and their interactions?"

## Stage 2: Identify relevant studies

### *Process of search strategy*

The study will consider all literature that reports on implementation of maternal and/or perinatal death audit published in English between 2004 and July 2018 from low and middle-income countries. The start year is selected to coincide with the first World Health Organization maternal death review guideline.[15]

We will include both quantitative and qualitative research studies. Peer-review publications will be primary sources but other published and unpublished (grey) literature such as reviews, reports, and commentaries will be also taken into consideration.

An initial limited search of three online databases, which are relevant to our topic, will be undertaken using Google Scholar and PubMed to pilot the search strategy terms. Medical subject heading terms from PubMed will be used at the start to determine the words used to search in PubMed. We will combine search terms focused on maternal mortality, perinatal mortality, audit/review systems, and attributes of audit/review systems (search strategy found in Supplementary File 2).

After the initial search, we will analyze the text words contained in the title and abstract of retrieved articles, and of the index terms used to describe the articles. Revisions of our search strategy will be



For peer review only - http://bmjopen.bmj.com/site/about/guidelines.xhtml

considered based on the findings of the initial search and incorporate additional keywords, sources and search terms as appropriate. A second search using all the identified keywords and the index terms specific to each database will be undertaken across accessible databases and websites. The search will then be performed using the following additional electronic databases and online search engines: PubMed, CINAHL, SCOPUS, Web of Science, JSTOR, LILACS, the WHO Library, Maternal Death Surveillance and Response Action Network, and Google.

The reference lists of all identified reports and articles will be searched for additional studies. All identified studies will be added into EndNote software and duplicate citations will be removed. We may contact the authors of primary studies or reviews for further information if necessary to provide clarity or to access additional information.

Finally, we will consult with experts in the field, including members of the World Health Organization's MPDSR Technical Working Group, to ensure we have identified all relevant literature (published and grey).

### *Characteristics of criteria*

Table 2 provides the inclusion criteria for this scoping review. For the concept component, we will only include literature that focuses on maternal or perinatal death reviews; thus excluding verbal autopsy or community death reviews, near-miss reviews, or confidential enquiries into maternal deaths. We also will exclude literature that does not specifically describe influencers of M/PDSR implementation. For example, some studies focus on the results of the audit data such as assessing cases of pre-eclampsia. If the article does not include factors exploring the implementation process, it will not be included.

**Table 2: Inclusion criteria for the scoping review**

| Components | Application to this scoping review |
|---|---|
| Concept component | • All forms of maternal and perinatal death review including obstetric audit, MPDSR, MDSR, MDR<br>• Limited to studies or perspectives that identify factors that influence the implementation process |
| Context component | • Limited to low and middle income countries listed by the World Bank in 2018. |

## Stage 3: Study selection

### *Process of screening and data extraction*

Two reviewers will independently screen titles and then abstracts to check for relevance to the review. The reviewers will regularly meet during the screening process to discuss their selection of articles and to refine screening, if needed. In the cases where abstracts are not available, the full text will be screened. All discrepancies between reviewers will be resolved by a third party.

Using the same process, the same reviewers will subsequently screen remaining full texts. All discrepancies between reviewers will be resolved by an independent third party.

For peer review only



For peer review only - http://bmjopen.bmj.com/site/about/guidelines.xhtml

## Stage 4: Data collection

A data collection instrument will be developed by the research team according to the JBI guidance. The extracted data will include study characteristics (e.g., type of reference such as article, report, study population, setting, study time period, study objective, study design). We will also consider the level or cultural factors addressed i.e. policies, law and rhetoric (macro level), internalised routine practice at the sub-national and facility levels (meso level), and individual behaviour change (micro level).[6]

For describing implementation of M/PDSR, extracted data will be based on the constructs identified in the conceptual framework. The draft data extraction components are provided in Supplementary File 3. We will pilot test the data extraction tool during a workshop and agree on any revisions. The same team members who will undertake the screening process will extract data from the selected articles. The team will engage in weekly meetings to discuss any issues or questions relating to the extraction process; decisions on extraction process will be documented. The charting table may be updated if other additional unforeseen data are identified as extraction moves forward.

## Stage 5: Data summary and synthesis of results

The review decision process will be reported using an adapted "Preferred Reporting Items for Systematic Reviews and Meta-Analyses (PRISMA)" extension for scoping reviews flow diagram.[45] Data analysis will involve qualitative thematic and content analysis linked to the conceptual framework.[46]

## Stage 6: Consultation

Stakeholders will be engaged throughout the scoping review from helping to identify literature, to providing input on the conceptual framework and reviewing the findings to support interpretation. Consultations will be targeted at experts serving on the World Health Organization's MPDSR Technical Working Group, as well as the Countdown to 2030 Drivers Technical Working Group. Other experts will be identified through a snowballing approach.

## Patient and Public Involvement

Given this is a protocol for a scoping review, patients and public were not involved in the design or research of the study.

## Proposed timeline

The process for conceptualizing the scoping review, including the framework, began in April 2018. From September 2018 to March 2019, we began the consultation process with key stakeholders as well as the screening process. Data collection began in April 2019. We expect the scoping review will be completed in the first quarter of 2020.

**DISSEMINATION AND ETHICS**

This study will contribute to the work of the Countdown to 2030 Drivers Group, which explores key contextual drivers for equitable and effective coverage of maternal and child health interventions as well as the MPDSR Technical Working Group co-led by UNFPA, UNICEF and the World Health Organization, which is tasked with advancing implementation of the intervention. This scoping review seeks to contribute specifically to the understanding how implementation of M/PDSR can drive quality



For peer review only - http://bmjopen.bmj.com/site/about/guidelines.xhtml

For peer review only

improvements in service delivery responses to women and children's health. Limitations of the study include the parameters of the search criteria, notably around language (English only) and time span (from 2004-July 2018) and search process (e.g. not all grey literature will be identified). Because of these limitations, some literature or components of the identified literature may not be included in the results of this scoping review. Ethics approval is not required since the scoping review methodology consists of reviewing and collecting data from publicly available sources. We plan to publish the results of the scoping review in an academic journal as well as present to key stakeholders through various forum (i.e. webinars, conferences, meetings). Consultation with key stakeholder groups will further guide dissemination efforts.

For peer review only



For peer review only - http://bmjopen.bmj.com/site/about/guidelines.xhtml

## Contributors

MK conceived of the idea, developed the research question and study methods and contributed meaningfully to the drafting and editing; she also approved the final manuscript. AG, DRW, PW, PW aided in developing the research question and study methods, contributed meaningfully to the drafting and editing, and approved the final manuscript.

## Funding

Asha George and Mary Kinney are supported by the South African Research Chair's Initiative of the Department of Science and Technology and National Research Foundation of South Africa (Grant No 82769), the South African Medical Research Council and the Countdown 2030 project funded by the Bill and Melinda Gates Foundation. Any opinion, finding and conclusion or recommendation expressed in this material is that of the author and funders do not accept any liability in this regard.

## Competing interests

None declared.

## Provenance and peer review

Not commissioned; externally peer reviewed.

## Data sharing statement

No additional data are available.

**Figure 1: Conceptual framework for implementation of M/PDSR**

For peer review only

REFERENCES

1. Bhutta ZA, Salam RA, Lassi ZS, et al. Approaches to improve quality of care (QoC) for women and newborns: conclusions, evidence gaps and research priorities. *Reprod Health* 2014;11 Suppl 2:S5. doi: 10.1186/1742-4755-11-S2-S5
2. Montagu D, Sudhinaraset M, Diamond-Smith N, et al. Where women go to deliver: understanding the changing landscape of childbirth in Africa and Asia. *Health policy and planning* 2017;32(8):1146-52. doi: 10.1093/heapol/czx060
3. Independent Expert Review Group. A post-2015 vision. The Third Report of the Independent Expert Review Group on Information and Accountability for Women's and Children's Health. Geneva: World Health Organization, 2014.
4. Hounton S, De Bernis L, Hussein J, et al. Towards elimination of maternal deaths: maternal deaths surveillance and response. *Reprod Health* 2013;10:1. doi: 10.1186/1742-4755-10-1
5. Every Woman Every Child. The Global Strategy for Women's, Children's and Adolescents' Health. New York, NY: Every Woman Every Child, 2015.
6. Lewis G. The cultural environment behind successful maternal death and morbidity reviews. *Bjog* 2014;121 Suppl 4:24-31. doi: 10.1111/1471-0528.12801
7. Heiby JR, Armbruster D, Jacobs TA. Better care for every patient, every time: improving quality in low health systems. *Bjog* 2014;121 Suppl 4:4-7. doi: 10.1111/1471-0528.12903
8. WHO. Making every baby count: audit and review of stillbirths and neonatal deaths. Geneva: World Health Organization, 2016.
9. Kerber KJ, Mathai M, Lewis G, et al. Counting every stillbirth and neonatal death through mortality audit to improve quality of care for every pregnant woman and her baby. *BMC Pregnancy Childbirth* 2015;15 Suppl 2:S9. doi: 10.1186/1471-2393-15-S2-S9
10. Pattinson R, Kerber K, Waiswa P, et al. Perinatal mortality audit: counting, accountability, and overcoming challenges in scaling up in low- and middle-income countries. *Int J Gynaecol Obstet* 2009;107 Suppl 1:S113-21, S21-2. doi: S0020-7292(09)00362-2 [pii] 10.1016/j.ijgo.2009.07.011 [published Online First: 2009/10/10]
11. Ivers N, Jamtvedt G, Flottorp S, et al. Audit and feedback: effects on professional practice and healthcare outcomes. *Cochrane Database Syst Rev* 2012;6:CD000259. doi: 10.1002/14651858.CD000259.pub3
12. MDSR Action Network Evidence 4 Action; 2017 [Available from: http://mdsr-action.net/ accessed 5 September 2017.
13. WHO. Global Monitoring of Implementation of Maternal Death Surveillance and Response (MDSR) 2017 [Available from: http://www.who.int/maternal_child_adolescent/epidemiology/maternal-death-surveillance/global-monitoring/en/ accessed 5 September 2017.
14. WHO. Time to respond: a report on the global implementation of maternal death surveillance and response (MDSR). Geneva: World Health Organization, 2016.
15. WHO. Beyond the numbers: Reviewing maternal deaths and complications to make pregnancy safer. Geneva: World Health Organization, 2004.
16. WHO. Maternal death surveillance and response: technical guidance. Geneva: World Health Organization, 2013.
17. Martin Hilber A, Blake C, Bohle LF, et al. Strengthening accountability for improved maternal and newborn health: A mapping of studies in Sub-Saharan Africa. *Int J Gynaecol Obstet* 2016;135(3):345-57. doi: 10.1016/j.ijgo.2016.09.008



For peer review only - http://bmjopen.bmj.com/site/about/guidelines.xhtml

18. Smith H, Ameh C, Roos N, et al. Implementing maternal death surveillance and response: a review of lessons from country case studies. *BMC Pregnancy Childbirth* 2017;17(1):233. doi: 10.1186/s12884-017-1405-6
19. Akachi Y, Kruk ME. Quality of care: measuring a neglected driver of improved health. *Bull World Health Organ* 2017;95(6):465-72. doi: 10.2471/BLT.16.180190
20. Persson LA. Bridging the quality chasm in maternal, newborn, and child healthcare in low- and middle-income countries. *PLoS medicine* 2017;14(12):e1002465. doi: 10.1371/journal.pmed.1002465
21. Topp SM. The Lancet Global Health Commission on High Quality Health Systems-where's the complexity? *The Lancet Global health* 2017;5(6):e571. doi: 10.1016/S2214-109X(17)30176-6
22. Kruk ME, Marchant T, Doubova S, et al. The Lancet Global Health Commission on High Quality Health Systems-where's the complexity? - Authors' reply. *The Lancet Global health* 2017;5(6):e572. doi: 10.1016/S2214-109X(17)30178-X
23. Hulscher ME, Schouten LM, Grol RP, et al. Determinants of success of quality improvement collaboratives: what does the literature show? *BMJ quality & safety* 2013;22(1):19-31. doi: 10.1136/bmjqs-2011-000651
24. Davidoff F, Dixon-Woods M, Leviton L, et al. Demystifying theory and its use in improvement. *BMJ quality & safety* 2015;24(3):228-38. doi: 10.1136/bmjqs-2014-003627
25. Bauer MS, Damschroder L, Hagedorn H, et al. An introduction to implementation science for the non-specialist. *BMC Psychol* 2015;3:32. doi: 10.1186/s40359-015-0089-9
26. Langley A, Denis JL. Beyond evidence: the micropolitics of improvement. *BMJ quality & safety* 2011;20 Suppl 1:i43-6. doi: 10.1136/bmjqs.2010.046482
27. Nilsen P. Making sense of implementation theories, models and frameworks. *Implement Sci* 2015;10:53. doi: 10.1186/s13012-015-0242-0
28. George A, Scott K, Garimella S, et al. Anchoring contextual analysis in health policy and systems research: A narrative review of contextual factors influencing health committees in low and middle income countries. *Soc Sci Med* 2015;133:159-67. doi: 10.1016/j.socscimed.2015.03.049
29. WHO. Every Newborn: An action plan to end preventable newborn deaths. Geneva: World Health Organization, 2014.
30. WHO. Strategies toward ending preventable maternal mortality (EPMM). Geneva: World Health Organization, 2015.
31. Kruk ME, Pate M, Mullan Z. Introducing The Lancet Global Health Commission on High-Quality Health Systems in the SDG Era. *The Lancet Global health* 2017;5(5):e480-e81. doi: 10.1016/S2214-109X(17)30101-8
32. Nilsen P, Stahl C, Roback K, et al. Never the twain shall meet?--a comparison of implementation science and policy implementation research. *Implement Sci* 2013;8:63. doi: 10.1186/1748-5908-8-63
33. The Centre for Research in Implementation Science and Prevention (CRISP). Dissemination & implementation models in health research & practice 2015 [Available from: http://dissemination-implementation.com/index.aspx.
34. Kitson AL, Rycroft-Malone J, Harvey G, et al. Evaluating the successful implementation of evidence into practice using the PARiHS framework: theoretical and practical challenges. *Implement Sci* 2008;3:1. doi: 10.1186/1748-5908-3-1
35. Damschroder LJ, Aron DC, Keith RE, et al. Fostering implementation of health services research findings into practice: a consolidated framework for advancing implementation science. *Implement Sci* 2009;4:50. doi: 10.1186/1748-5908-4-50



For peer review only - http://bmjopen.bmj.com/site/about/guidelines.xhtml

36. Pfadenhauer LM, Gerhardus A, Mozygemba K, et al. Making sense of complexity in context and implementation: the Context and Implementation of Complex Interventions (CICI) framework. *Implement Sci* 2017;12(1):21. doi: 10.1186/s13012-017-0552-5
37. May CR, Johnson M, Finch T. Implementation, context and complexity. *Implement Sci* 2016;11(1):141. doi: 10.1186/s13012-016-0506-3
38. Chambers DA, Glasgow RE, Stange KC. The dynamic sustainability framework: addressing the paradox of sustainment amid ongoing change. *Implement Sci* 2013;8:117. doi: 10.1186/1748-5908-8-117
39. Murray E, Treweek S, Pope C, et al. Normalisation process theory: a framework for developing, evaluating and implementing complex interventions. *BMC medicine* 2010;8:63. doi: 10.1186/1741-7015-8-63
40. George A, al. e. Lenses and levels: the why, what and how of measuring health system drivers of women's, children's and adolescents' health with a governance focus. *BMJ global health* 2019 (in press)
41. Tricco AC, Lillie E, Zarin W, et al. A scoping review on the conduct and reporting of scoping reviews. *BMC medical research methodology* 2016;16:15. doi: 10.1186/s12874-016-0116-4
42. Arksey H, HO'Malley L. Scoping studies: towards a methodological framework. *Int J Soc Res Methodol* 2005;8(1):19-32. doi: 10.1080/1364557032000119616
43. Levac D, Colquhoun H, O'Brien KK. Scoping studies: advancing the methodology. *Implement Sci* 2010;5:69. doi: 10.1186/1748-5908-5-69
44. Joanna Briggs Institute Reviewer's Manual: 2015 edition. Adelaide, SA: Joanna Briggs Institute, 2015.
45. Tricco AC, Lillie E, Zarin W, et al. PRISMA Extension for Scoping Reviews (PRISMA-ScR): Checklist and Explanation. *Annals of internal medicine* 2018;169(7):467-73. doi: 10.7326/M18-0850
46. Vaismoradi M, Turunen H, Bondas T. Content analysis and thematic analysis: Implications for conducting a qualitative descriptive study. *Nurs Health Sci* 2013;15(3):398-405. doi: 10.1111/nhs.12048

For peer review only



For peer review only - http://bmjopen.bmj.com/site/about/guidelines.xhtml

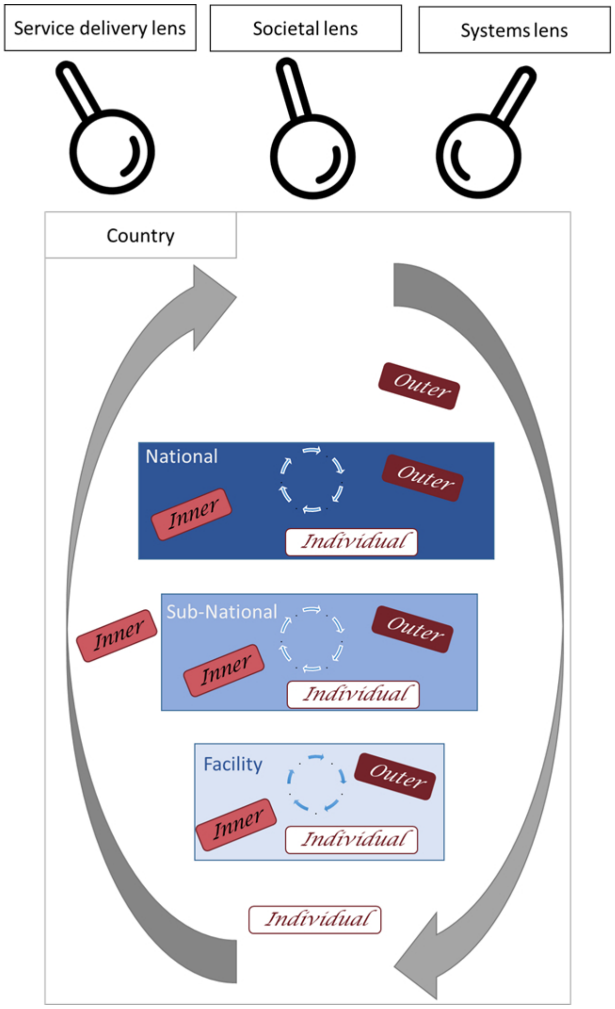


Figure 1: Conceptual framework for implementation of M/PDSR

90x89mm (300 x 300 DPI)

Supplementary Files for *Implementation of maternal and perinatal death reviews: a scoping review*

**SUPPLEMENTARY FILE 1**

**Table S1.1: Frameworks considered when developing the conceptual frameworks for the scoping review**

| Framework | Level | Characteristic | Pros - why we considered it | Cons – why we decided not to use it |
|---|---|---|---|---|
| Context and Implementation of Complex Intervention - CICI Framework (Pfadenhauer) [1] | Macro, Meso, Micro | Comprises three dimensions which interact: Context (i.e., geographical, epidemiological, socio-cultural, socio-economic, ethical, legal, political); implementation (i.e., implementation theory, process, strategies, agents and outcomes); setting (specific physical location). | A determinant framework that seeks to conceptualise, describe and understand the multiple influences on implementation outcomes; Enables understanding of implementation process at multiple levels across different factors; Provides data extraction tools for qualitative and quantitative reviews | It focuses on the operationalization of context, setting and implementation *not on the intervention itself.* Yet we wanted to understand more about the how the intervention is working and being implemented. |
| Dynamic sustainability framework (Chambers) [2] | Macro, Meso, | Considers the intervention, the context and the broader ecological system over time | A new paradigm to consider the long-term use and ongoing improvement of interventions; posits that ongoing QI is the aim | Does not provide enough focus on the implementation process now – rather would allow for exploring implementation of audit over time to see if process leads to change (which is not purpose of scoping review) |
| Promoting Action on Research Implementation in Health Services - PARiHS (Kitson) [3] | Meso | Considers the interactions between three key elements: Evidence, Context, Facilitation | It argues that successful implementation (SI) of evidence into practice had as much to do with the context or setting where the new evidence was being | Focuses on knowledge translation component of implementation. Many of the constructs do not relate to what we think would be needed to |



For peer review only - http://bmjopen.bmj.com/site/about/guidelines.xhtml

| | | | | |
|---|---|---|---|---|
| | | | introduced and how it was introduced | understand the MPDSR process |
| Consolidated Framework for Implementation Research - CFIR (Damschroder) [4] | Macro, Meso, Macro | Comprises five major domains and considers their interactions: the intervention, inner and outer setting, the individuals involved, and the process by which implementation is accomplished | Enables understanding of different levels and different factors that influence implementation; it includes the intervention outcome but also allows for understanding process as well as influencing factors at different levels | Not all constructs are applicable to MPDSR implementation and some elements of what we seek to understand might be missing |
| Normalization Process Theory (May) [5] | Meso, Micro | Comprises four constructs: Coherence, Cognitive Participation, Collective Action, and Reflexive Monitoring | it explores the fluid, dynamic and interactive processes between context, actors and objects Investigates the process by which an intervention becomes embedded | Does not include enough factors that should be considered for understanding implementation factors (focuses only on meso, micro level) |

For peer review only

**SUPPLEMENTARY FILE 2: SEARCH STRATEGY**

Search terms used for search engines from January 2004 to July 2018:

("maternal mortality" OR "perinatal death" OR "maternal death" OR "perinatal mortality" OR "fetal mortality" OR "stillbirth") **AND** ("audit" OR "surveillance and response")

**Table 2.1 Concept and search terms included**

| Concept | Search terms |
|---|---|
| Maternal & Perinatal death | maternal mortality, maternal death, stillbirth, fetal mortality, perinatal death, perinatal mortality |
| Audit | Audit, surveillance and response |

For peer review only



For peer review only - http://bmjopen.bmj.com/site/about/guidelines.xhtml

**SUPPLEMENTARY FILE 3: DRAFT DATA EXTRACTION FORM**

| | | |
|---|---|---|
| | Reviewer assigned | |
| | Inclusion | Yes<br>No |
| **Reference details** | Ref ID | |
| | Author | |
| | Title | |
| | Year | |
| | Abstract | |
| | Journal | |
| | Volume | |
| | Issue | |
| | Pages | |
| | URL | |
| | Source | Database<br>Online search<br>Consultation |
| Background to reference | Country of first affiliation | |
| | Country of Study | Name country<br>Multiple<br>N/A |
| | Country Setting | Upper MI<br>Lower MI<br>LIC<br>LMIC<br>Not specified (add to notes) |
| | Region of World | Sub-Saharan Africa<br>East Asia & Pacific<br>Latin America & Caribbean<br>South Asia<br>Europe & Central Asia<br>Middle East & North Africa<br>International |
| | Organization Type of authors | Government<br>NGO<br>University/academic institution<br>Independent<br>Mixed including government<br>Mixed not including government<br>Other (add note) |
| | Funder type (sector) | Government<br>Multilateral (e.g. WHO, World Bank, UN)<br>Bilateral (e.g. USAID, DIFD, IDRC)<br>Foundation (e.g. BMGF, Rockefeller)<br>Mixed includes government |



For peer review only - http://bmjopen.bmj.com/site/about/guidelines.xhtml

| | | |
|---|---|---|
| | | Mixed does not include government<br>No funding received |
| | Notes | |
| Content of reference | Focus of the audit | Maternal & perinatal<br>Maternal<br>Perinatal |
| | Notes | |
| | History/timeline | Yes<br>No |
| | Notes | |
| | Scale | National<br>Subnational<br>Selected Facilities<br>Combination Of Levels<br>Global<br>Multicountry<br>Other |
| | Notes | |
| Reference type | Document type | Academic journal article<br>Academic journal commentary<br>Academic review<br>Grey lit online<br>Grey lit not online<br>other |
| | Methods section | Yes<br>No |
| | Research Design | Quantitative<br>Qualitative<br>Mixed<br>NA |
| | Level of Study | Macro<br>Meso<br>Micro<br>Combination |
| | Notes | |
| Domain 1: Intervention/MPDSR | Executing audit | Described<br>Not described |
| | Notes | |
| | Cost & funding | Described<br>Not described |
| | Notes | |
| | Intervention source | Described<br>Not described |
| | Notes | |
| | Evidence strength & quality | Described<br>Not described |



For peer review only - http://bmjopen.bmj.com/site/about/guidelines.xhtml

| | | |
|---|---|---|
| | Notes | |
| | Relative advantage | Described<br>Not described |
| | Notes | |
| | Trialability | Described<br>Not described |
| | Notes | |
| | Reflectivity | Described<br>Not described |
| | Notes | |
| | Adaptability | Described<br>Not described |
| | Notes | |
| | Complexity | Described<br>Not described |
| | Notes | |
| Domain 2: Outer Setting | Policy and planning | Described<br>Not described |
| | Notes | |
| | Resource flows | Described<br>Not described |
| | Notes | |
| | Linkages to other actors | Described<br>Not described |
| | Notes | |
| | Pressure | Described<br>Not described |
| | Notes | |
| | Community links | Described<br>Not described |
| | Notes | |
| | Cosmopolitanism | Described<br>Not described |
| | Notes | |
| Domain 3: Inner Setting | Readiness to implement | Described<br>Not described |
| | Notes | |
| | Structural characteristics of social architecture | Described<br>Not described |
| | Notes | |
| | Incentive/rewards | Described<br>Not described |
| | Notes | |
| | Networks & communication | Described<br>Not described |

| | | |
|---|---|---|
| | Notes | |
| | Culture | Described<br>Not described |
| | Notes | |
| | Implementation climate | Described<br>Not described |
| | Notes | |
| | Agents of change | Described<br>Not described |
| | Notes | |
| Domain 4: Individuals | Tech skills & knowledge | Described<br>Not described |
| | Notes | |
| | Individual motivation, Self-efficacy | Described<br>Not described |
| | Notes | |
| | Individual commitment | Described<br>Not described |
| | Notes | |
| | Individual orientation | Described<br>Not described |
| | Notes | |
| | Individual state of change | Described<br>Not described |
| | Notes | |

**REFERENCES**

1. Pfadenhauer LM, Gerhardus A, Mozygemba K, Lysdahl KB, Booth A, Hofmann B et al. Making sense of complexity in context and implementation: the Context and Implementation of Complex Interventions (CICI) framework. Implement Sci. 2017; 12(1):21.
2. Chambers DA, Glasgow RE,Stange KC. The dynamic sustainability framework: addressing the paradox of sustainment amid ongoing change. Implement Sci. 2013; 8:117.
3. Kitson AL, Rycroft-Malone J, Harvey G, McCormack B, Seers K,Titchen A. Evaluating the successful implementation of evidence into practice using the PARiHS framework: theoretical and practical challenges. Implement Sci. 2008; 3:1.
4. Damschroder LJ, Aron DC, Keith RE, Kirsh SR, Alexander JA,Lowery JC. Fostering implementation of health services research findings into practice: a consolidated framework for advancing implementation science. Implement Sci. 2009; 4:50.
5. Murray E, Treweek S, Pope C, MacFarlane A, Ballini L, Dowrick C et al. Normalisation process theory: a framework for developing, evaluating and implementing complex interventions. BMC medicine. 2010; 8:63.



For peer review only - http://bmjopen.bmj.com/site/about/guidelines.xhtml
